# Gastrointestinal toxicity among patients taking selective COX‐2 inhibitors or conventional NSAIDs, alone or combined with proton pump inhibitors: a case–control study

**DOI:** 10.1002/pds.4183

**Published:** 2017-03-31

**Authors:** Mohammad Bakhriansyah, Patrick C. Souverein, Anthonius de Boer, Olaf H. Klungel

**Affiliations:** ^1^ Division of Pharmacoepidemiology and Clinical Pharmacology Utrecht Institute of Pharmaceutical Sciences The Netherlands; ^2^ Department of Pharmacology, Medical Faculty Lambung Mangkurat University Banjarmasin Indonesia

**Keywords:** gastrointestinal toxicity, perforation, ulcers, bleeding, conventional NSAIDs, selective COX‐2 inhibitors, proton pump inhibitors

## Abstract

**Purpose:**

To assess the risk of gastrointestinal perforation, ulcers, or bleeding (PUB) associated with the use of conventional nonsteroidal anti‐inflammatory drugs (NSAIDs) with proton pump inhibitors (PPIs) and selective COX‐2 inhibitors, with or without PPIs compared with conventional NSAIDs.

**Methods:**

A case–control study was performed within conventional NSAIDs and/or selective COX‐2 inhibitors users identified from the Dutch PHARMO Record Linkage System in the period 1998–2012. Cases were patients aged ≥18 years with a first hospital admission for PUB. For each case, up to four controls were matched for age and sex at the date a case was hospitalized (index date). Logistic regression analysis was used to calculate odds ratios (ORs).

**Results:**

At the index date, 2634 cases and 5074 controls were current users of conventional NSAIDs or selective COX‐2 inhibitors. Compared with conventional NSAIDs, selective COX‐2 inhibitors with PPIs had the lowest risk of PUB (adjusted OR 0.51, 95% confidence interval [CI]: 0.35–0.73) followed by selective COX‐2 inhibitors (adjusted OR 0.66, 95%CI: 0.48–0.89) and conventional NSAIDs with PPIs (adjusted OR 0.79, 95%CI: 0.68–0.92). Compared with conventional NSAIDs, the risk of PUB was lower for those aged ≥75 years taking conventional NSAIDs with PPIs compared with younger patients (adjusted interaction OR 0.79, 95%CI: 0.64–0.99). However, those aged ≥75 years taking selective COX‐2 inhibitors, the risk was higher compared with younger patients (adjusted interaction OR 1.22, 95%CI: 1.01–1.47).

**Conclusions:**

Selective COX‐2 inhibitors with PPIs, selective COX‐2 inhibitors, and conventional NSAIDs with PPIs were associated with lower risks of PUB compared with conventional NSAIDs. These effects were modified by age. © 2017 The Authors. *Pharmacoepidemiology & Drug Safety* Published by John Wiley & Sons Ltd.

## Introduction

Nonsteroidal anti‐inflammatory drugs (NSAIDs) are extensively used to treat pain‐related musculoskeletal diseases such as osteoarthritis, rheumatoid arthritis, and chronic low back pain.^1^, ^2^, ^3^ Conventional NSAIDs inhibit the cyclooxygenase (COX) isoenzymes, COX‐1 and COX‐2, while the selective COX‐2 inhibitors mainly inhibit the latter.^4^

Two meta‐analyses of clinical trials showed that conventional NSAIDs increase the risk of gastrointestinal (GI) complications.^5^, ^6^ Although selective COX‐2 inhibitors have a lower risk of GI toxicity than conventional NSAIDs, a meta‐analysis of clinical trials showed that celecoxib still increases the risk of GI toxicity compared with placebo.^7^

Several evidence‐based strategies are implemented to lower the risk of GI adverse events when a NSAID is needed, such as substitution of conventional NSAIDs for selective COX‐2 inhibitors or coadministration of proton pump inhibitors (PPIs) with conventional NSAIDs.^8^, ^9^, ^10^, ^11^ When conventional NSAIDs are combined with PPIs, the risk of symptomatic GI ulcers is lower than with conventional NSAIDs alone,^11^, ^12^ in particular for patients with risk factors for GI complications and long‐term use.^13^ Furthermore, a meta‐analysis of clinical trials demonstrated that the risk of upper GI toxicity for the combined treatment of a conventional NSAID and a PPI is similar for selective COX‐2 inhibitors alone.^14^

Another strategy to reduce GI toxicity is by combining selective COX‐2 inhibitors with PPIs.^15^ Several studies showed that this combination is associated with a lower risk of GI adverse events compared with conventional NSAIDs^16^, ^17^, ^18^ or selective COX‐2 inhibitors alone.^19^, ^20^

Compared with younger users, elderly aged ≥75 years taking ibuprofen with omeprazole showed a higher risk of recurrent ulcers^21^ and a combination of celecoxib and a PPI was more beneficial to decrease the risk of GI hospitalization with celecoxib as a comparator.^22^ Male gender is also associated with a higher risk of GI adverse events among conventional NSAIDs users.^23^

As presented earlier, there is a large body of evidence about the GI protective strategies when patients with an increased risk of GI problems are in need of a NSAID. Still, it was shown in an observational study that in clinical practice, >58% of NSAID users with an increased risk for GI problems do not receive a gastroprotective strategy.^24^ This undertreatment might be partly explained by the fact that there is no clear recommendation when to use which strategy. It is probably related to the fact that the relative effects of the different GI protective strategies are largely unknown.

There have been many studies published in which the GI safety of conventional NSAIDs or selective COX‐2 inhibitors, alone or combined with a PPI, were compared. However, these different GI protective strategies were never evaluated in one study together. We, therefore, conducted a study comparing the relative risks of PUB for selective COX‐2 inhibitors with PPIs, selective COX‐2 inhibitors alone, and conventional NSAIDs with PPIs versus conventional NSAIDs alone, and to identify whether age, sex, and availability of PPIs as over‐the‐counter (OTC) drug modify these risk estimates.

## Methods

### Data source

Data were obtained from the Dutch PHARMO Record Linkage System (PHARMO RLS) from January 1998 until December 2012. This is a population‐based network of healthcare databases combining data from different healthcare settings in the Netherlands, such as hospitalization database, out‐patient and in‐patient pharmacy, and general practitioner database. More than 4 million (25%) inhabitants in the Netherlands have participated in this database. Patient's histories include detailed information about all drugs dispensed by date of dispensing, type of prescriber, dose, and duration of use, surgical procedure, discharge diagnosis, cost, and other administrative information.^25^, ^26^

### Study design and population

We conducted a case–control study in subjects who had ever used conventional NSAIDs and/or selective COX‐2 inhibitors. Cases were patients aged ≥18 years at first hospital admission with a primary discharge diagnosis of GI toxicity defined as PUB in the GI tract (The International Classification of Diseases, Ninth Revision, Clinical Modification codes 531, 532, and 533). The date of hospital admission was defined as the index date. Potential controls were patients without any diagnoses of GI toxicity prior to and at the index date of the case to which they were matched. For each case, up to four controls were matched on year of birth and sex at the index date.

### Exposure definition

All prescriptions for conventional NSAIDs, selective COX‐2 inhibitors, and PPIs before the index date were identified. Exposure classification was based on the use of conventional NSAIDs (Anatomical Therapeutic Chemical codes M01AA, M01AB, M01AC, M01AE, M01AG, or M01AX) alone or combined with PPIs (A02BC), or selective COX‐2 inhibitors (M01AH) alone or combined with PPIs. Patients were classified as current users when the theoretical end date of the last prescription ended after the index date. We allowed the gap by a half duration of the previous prescription between the end date of the prescription and the start date of the following one. We included only current users of conventional NSAIDs or selective COX‐2 inhibitors (without or with PPIs) in the analysis. Patients who had both conventional NSAIDs and selective COX‐2 inhibitors at the index date were excluded.

### Potential confounders

Potential confounders taken into account were age, sex, and concomitant drug use on the index date, including antacids (Anatomical Therapeutic Chemical code A02A), histamine‐2 receptor antagonists (A02BA), phenprocoumon (B01AA04), acenocoumarol (B01AA07), clopidogrel (B01AC04), acetylsalicylic acid (B01AC06), dipyridamole (B01AC07), prasugrel (B01AC22), glucocorticoids (H02AB), and selective serotonin reuptake inhibitors (N06AB). Potential confounders measured in the year prior to the index date were a history of conventional NSAIDs, selective COX‐2 inhibitors, antacid, histamine‐2 receptor antagonists, or PPI use.

### Data analyses

Logistic regression was used to estimate crude and adjusted odds ratios (ORs) and 95% confidence intervals (95%CI) of the risk of PUB associated with the current use of conventional NSAIDs with PPIs, selective COX‐2 inhibitors alone, or selective COX‐2 inhibitors with PPIs compared with conventional NSAIDs alone. We also evaluated the interaction by age, sex, and availability of PPIs as OTC drug by entering product terms in the model. Availability of PPIs as OTC drug was defined by the date when PPIs were first available as OTC drug in the Netherlands (February 2000). The synergy index (SI) was calculated to assess the risk and the significance of these interactions. The SI is defined as an interaction term between two variables. On the relative risk scale (multiplicative), this quantity measures whether the effect of both exposures together exceeds the product of the effects of the two exposures considered separately. If the SI >1, the interaction is said to be positive. In contrast, if the SI <1, the interaction is negative. A 95%CI of SI is used to define the significance of the interaction. All the analyses were carried out using IBM Statistic SPSS 23 and *p*‐values of <0.05 were considered statistically significant.

### Sensitivity analysis

For our main analysis, we defined current use if the index date fell within a time period of the last prescription of conventional NSAIDs or selective COX‐2 inhibitors. Patients who discontinued medication within 90 days prior to the index date were excluded. Because the gap between current and recent use was narrow, a sensitivity analysis was performed in which current users were defined as patients who discontinued medication in a time window of 90 days prior to the index date or were current users at the index date.

## Results

In the cohort, we identified 15 962 PUB cases and 62 683 age‐matched and sex‐matched controls among users of conventional NSAIDs and/or selective COX‐2 inhibitors within our 15‐year study period. Of those, 2634 cases and 5074 controls were current users of conventional NSAIDs or selective COX‐2 inhibitors (with or without PPIs) at the index date. By restricting to current users, the original matching ratio was not retained. Compared with controls, cases had more comorbidities determined by the number of concomitant drug use, namely, acid‐lowering drugs, vitamin K antagonists, platelet aggregation inhibitors, glucocorticoids, and selective serotonin reuptake inhibitors. The prevalence of drug use before the index date was also higher, for example, selective COX‐2 inhibitors and acid‐lowering drugs (Table 1).

**Table 1:** Baseline characteristics of cases with PUB and controls exposed to current use of conventional NSAIDs or selective COX‐2 inhibitors

		Cases (*n* = 2634)	Controls (*n* = 5074)	*p*‐value
Age	Mean (year ± SD)	68.75 ± 15.6	69.28 ± 14.6	0.135
Sex	Women, *n* (%)	1576 (59.8)	3084 (60.8)	0.420
Concomitant drug(s) use at the index date	Acid‐lowering drugs, *n* (%)†	164 (6.2)	187 (3.7)	0.000*
	Vitamin K antagonists, *n* (%)‡	399 (15.1)	244 (4.8)	0.000*
	Platelet aggregation inhibitors, *n* (%)§	707 (26.8)	999 (19.7)	0.000*
	Glucocorticoids, *n* (%)	188 (7.1)	234 (4.6)	0.000*
	Serotonin selective reuptake inhibitors, *n* (%)	132 (5.0)	205 (4.0)	0.048*
History of drug(s) use	Conventional NSAIDs, *n* (%)	192 (7.3)	502 (9.9)	0.000*
	Selective COX‐2 inhibitors, *n* (%)	409 (15.5)	619 (12.2)	0.000*
	Conventional NSAIDs + selective COX‐2 inhibitors, *n* (%)	0 (0.0)	0 (0.0)	NA
	Acid‐lowering drugs, *n* (%)¶	1444 (54.8)	2432 (47.9)	0.000*

NSAIDs, nonsteroidal anti‐inflammatory drugs; COX‐2, cyclooxygenase‐2; PUB, perforation, ulcers, or bleeding; NA, not applicable; SD, standard deviation.

† Acid‐lowering drugs (antacid and H2‐receptor antagonists).

‡ Vitamin K antagonists (phenprocoumon and acenocoumarol).

§ Platelet aggregation inhibitors (clopidogrel, acetyl salicylic acid, dipyridamole, and prasugrel).

¶ Acid‐lowering drugs (antacid, H2‐receptor antagonists, and proton pump inhibitors).

* Statistically significant (*p* < 0.05).

### Risk of PUB for current users of conventional NSAIDs or selective COX‐2 inhibitors, alone or combined with PPIs

Compared with conventional NSAIDs, selective COX‐2 inhibitors with PPIs were associated with a lower risk of PUB (adjusted OR 0.51, 95%CI: 0.35–0.73) followed by selective COX‐2 inhibitors (adjusted OR 0.66, 95%CI: 0.48–0.89) and conventional NSAIDs with PPIs (adjusted OR 0.79, 95%CI: 0.68–0.92) (Table 2). When we defined selective COX‐2 inhibitors alone as a reference group, the relative risks for conventional NSAIDs with PPIs and selective COX‐2 inhibitors with PPIs were not statistically different (adjusted OR 0.77, 95%CI: 0.55–1.07 and adjusted OR 1.21, 95%CI: 0.87–1.68, respectively) (table not shown).

**Table 2:** Odds ratios for PUB events among current users of conventional NSAIDs or selective COX‐2 inhibitors alone or combined with PPIs

Exposure	Cases (*n* = 2634)	Controls (*n* = 5074)	Crude OR (95%CI)	Adjusted OR† (95%CI)
Current use, *n* (%)				
Conventional NSAIDs − PPIs	1599 (60.7)	3013 (59.4)	1	1
Conventional NSAIDs + PPIs	775 (29.4)	1356 (26.7)	1.08 (0.97–1.20)	0.79 (0.68–0.92)*
Selective COX‐2 inhibitors − PPIs	179 (6.8)	487 (9.6)	0.69 (0.58–0.83)*	0.66 (0.48–0.89)*
Selective COX‐2 inhibitors + PPIs	81 (3.1)	218 (4.3)	0.70 (0.54–0.91)*	0.51 (0.35–0.73)*

NSAIDs, nonsteroidal anti‐inflammatory drugs; COX‐2, cyclooxygenase‐2; PPIs, proton pump inhibitors; OR, odd ratio; CI, confidence interval; PUB, perforation, ulcers, or bleeding.

† Adjusted for age, sex, concomitant drugs (acid‐lowering drugs, vitamin K antagonists, platelet aggregation inhibitors, glucocorticoids, and selective serotonin receptor inhibitors), and a history of drug use (conventional NSAIDs, selective COX‐2 inhibitors, and acid‐lowering drugs).

* Statistically significant (*p* < 0.05).

### Effect modification

For all age groups, our study revealed that conventional NSAIDs with PPIs, selective COX‐2 inhibitors alone, and selective COX‐2 inhibitors with PPIs decreased the relative risk of PUB compared with conventional NSAIDs alone as we found in our main analyses. Compared with younger patients, those aged ≥75 years taking conventional NSAIDs with PPIs had a lower risk (adjusted OR 0.69, 95%CI: 0.47–1.03 vs. adjusted OR 0.87, 95%CI: 0.73–1.04), but those aged ≥75 years taking selective COX‐2 inhibitors were associated with a higher risk (adjusted OR 0.88, 95%CI: 0.64–1.22 vs. adjusted OR 0.72, 95%CI: 0.63–0.83) with conventional NSAIDs alone as the comparator. These interactions were statistically significant (adjusted interaction OR 0.79, 95%CI: 0.64–0.99 for conventional NSAIDs with PPIs and adjusted interaction OR 1.22, 95%CI: 1.01–1.47 for selective COX‐2 inhibitors). Even though patients aged ≥75 years taking selective COX‐2 inhibitors with PPIs had a lower risk of PUB compared with younger patients (adjusted OR 0.71, 95%CI: 0.53–0.97 vs. adjusted OR 0.85, 95%CI: 0.75–0.97), the interaction was not statistically significant (adjusted interaction OR 0.84, 95%CI: 0.70–1.00) (Table 3).

**Table 3:** Effect modification of age toward the association between conventional NSAIDs or selective COX‐2 inhibitors alone or combined with PPIs and the risk of PUB

	Cases	Controls	Crude OR (95%CI)	Adjusted OR† (95%CI)	Crude SI (95%CI)	Adjusted SI† (95%CI)
Age 18–74 years, *n* (%)					0.83 (0.67–1.03)	0.79 (0.64–0.99)*
Conventional NSAIDs − PPIs	948 (68.4)	1820 (71.7)	1	1		
Conventional NSAIDs + PPIs	438 (31.6)	718 (28.3)	1.17 (1.02–1.35)*	0.87 (0.73–1.04)		
Age ≥75 years, *n* (%)						
Conventional NSAIDs − PPIs	651 (65.9)	1193 (65.2)	1	1		
Conventional NSAIDs + PPIs	337 (34.1)	638 (34.8)	0.97 (0.68–1.39)	0.69 (0.47–1.03)		
Age 18–74 years, *n* (%)					1.25 (1.04–1.50)	1.22 (1.01–1.47)*
Conventional NSAIDs − PPIs	948 (92.2)	1820 (87.7)	1	1		
Selective COX‐2 inhibitors − PPIs	72 (7.1)	255 (12.3)	0.74 (0.64–0.84)*	0.72 (0.63–0.83)*		
Age ≥75 years, *n* (%)						
Conventional NSAIDs − PPIs	651 (85.9)	1193 (83.7)	1	1		
Selective COX‐2 inhibitors − PPIs	107 (14.1)	232 (16.3)	0.93 (0.67–1.26)	0.88 (0.64–1.22)		
Age 18–74 years, *n* (%)					0.84 (0.70–1.00)*	0.84 (0.70–1.00)
Conventional NSAIDs − PPIs	948 (95.4)	1820 (94.9)	1	1		
Selective COX‐2 inhibitors + PPIs	46 (4.6)	97 (5.1)	0.97 (0.86–1.09)	0.85 (0.75–0.97)*		
Age ≥75 years, *n* (%)						
Conventional NSAIDs − PPIs	651 (94.9)	1193 (90.8)	1	1		
Selective COX‐2 inhibitors + PPIs	35 (5.1)	121 (9.2)	0.81 (0.60–1.09)	0.71 (0.53–0.97)*		

NSAIDs, nonsteroidal anti‐inflammatory drugs; COX‐2, cyclooxygenase‐2; PPIs, proton pump inhibitors; OR, odd ratio; CI, confidence interval; SI, synergy index; PUB, perforation, ulcers, or bleeding.

† Adjusted for sex, concomitant drugs (acid‐lowering drugs, vitamin K antagonists, platelet aggregation inhibitors, glucocorticoids, and selective serotonin receptor inhibitors), and a history of drug use (conventional NSAID, selective COX‐2 inhibitors, and acid‐lowering drugs).

* Statistically significant (*p* < 0.05).

In contrast to age, our study indicated that sex did not modify the risk of PUB for conventional NSAIDs plus PPIs or selective COX‐2 inhibitors (with or without PPIs) (Table 4), and availability of PPIs as OTC drug did not modify the risk of PUB for conventional NSAIDs with PPIs all compared with conventional NSAIDs alone (Appendix). The interaction between availability of PPIs as OTC drug and selective COX‐2 inhibitors (with or without PPIs) could not be determined because OTC PPIs have been available before the first selective COX‐2 inhibitors were introduced in the Netherlands in May 2000.^27^

**Table 4:** Effect modification of sex toward the association between conventional NSAIDs or selective COX‐2 inhibitors alone or combined with PPIs and the risk of PUB

	Cases	Controls	Crude OR (95%CI)	Adjusted OR† (95%CI)	Crude SI (95%CI)	Adjusted SI† (95%CI)
Women, *n* (%)					0.82 (0.66–1.01)	0.84 (0.67–1.05)
Conventional NSAIDs − PPIs	949 (68.0)	1757 (67.8)	1	1		
Conventional NSAIDs + PPIs	447 (32.0)	835 (32.2)	1.22 (1.03–1.44)*	0.89 (0.72–1.08)		
Men, *n* (%)						
Conventional NSAIDs − PPIs	650 (60.6)	1256 (62.8)	1	1		
Conventional NSAIDs + PPIs	328 (30.6)	521 (26.1)	1.00 (0.68–1.45)	0.75 (0.48–1.14)		
Women, *n* (%)					0.97 (0.80–1.17)	0.97 (0.80–1.19)
Conventional NSAIDs − PPIs	949 (88.8)	1756 (70.7)	1	1		
Selective COX‐2 inhibitors − PPIs	120 (11.2)	329 (29.3)	0.85 (0.73–0.99)*	0.82 (0.69–0.96)*		
Men, *n* (%)						
Conventional NSAIDs − PPIs	650 (91.7)	1256 (88.8)	1	1		
Selective COX‐2 inhibitors − PPIs	59 (8.3)	158 (11.2)	0.82 (0.58–1.14)	0.80 (0.55–1.14)		
Women, *n* (%)					0.97 (0.80–1.19)	1.02 (0.83–1.25)
Conventional NSAIDs − PPIs	949 (94.1)	1757 (91.5)	1	1		
Selective COX‐2 inhibitors + PPIs	60 (5.9)	163 (8.5)	0.90 (0.76–1.07)	0.77 (0.65–0.92)*		
Men, *n* (%)						
Conventional NSAIDs − PPIs	650 (96.9)	1256 (95.8)	1	1		
Selective COX‐2 inhibitors + PPIs	21 (3.1)	55 (4.2)	0.87 (0.61–1.27)	0.79 (0.54–1.49)		

NSAIDs, nonsteroidal anti‐inflammatory drugs; COX‐2, cyclooxygenase‐2; PPIs, proton pump inhibitors; OR, odd ratio; CI, confidence interval; SI, synergy index; PUB, perforation, ulcers, or bleeding.

† Adjusted for age, concomitant drugs (acid‐lowering drugs, vitamin K antagonists, platelet aggregation inhibitors, glucocorticoids, and selective serotonin receptor inhibitors), and a history of drug use (conventional NSAIDs, selective COX‐2 inhibitors, and acid‐lowering drugs).

* Statistically significant (*p* < 0.05).

### Sensitivity analysis

In our sensitivity analysis, we defined current users as patients who discontinued the medication within 90 days prior to the index date or were current users at the index date. Selective COX‐2 inhibitors with PPIs and selective COX‐2 inhibitors alone decreased the relative risk of PUB by 16% (adjusted OR 0.84, 95%CI: 0.62–1.13) and by 15% (adjusted OR 0.85, 95%CI: 0.67–1.06), respectively, compared with conventional NSAIDs. However, these relative risks were not statistically significant. Unexpectedly, conventional NSAIDs with PPIs significantly increased the risk by 25% (adjusted OR 1.25, 95%CI: 1.13–1.38) compared with conventional NSAIDs alone (Appendix).

## Discussion

This study demonstrated that compared with conventional NSAIDs, conventional NSAIDs with PPIs, selective COX‐2 inhibitors alone, and selective COX‐2 inhibitors with PPIs decreased the risk of PUB with 21%, 34%, and 49%, respectively. Furthermore, our study showed that in patients >75 years old the GI protective effect of conventional NSAIDs with PPIs and selective COX‐2 inhibitors with PPIs were higher than in patients <75. However, for selective COX‐2 inhibitors alone, this protective effect in the older age group unexpectedly appeared less. Sex and availability of PPIs as OTC drugs did not modify the effect of these gastroprotective strategies.

These results, which were obtained from one study, are consistent with several earlier studies in which the different contrasts were evaluated separately. Two systematic reviews of clinical trials showed that selective COX‐2 inhibitors or conventional NSAIDs with PPIs were associated with a lower risk of GI ulcers by 74% and 91%, respectively, compared with conventional NSAIDs.^8^, ^11^ Several observational studies also concluded that selective COX‐2 inhibitors with PPIs were associated with a 39–64% lower risk of upper GI complications compared with conventional NSAIDs.^16^, ^17^, ^18^

A meta‐analysis of clinical trials also showed that the relative risk of upper GI adverse events for conventional NSAIDs with PPIs was comparable with selective COX‐2 inhibitors.^14^ Furthermore, two clinical trials showed that the risks of GI ulcers were reduced by 8.9–15.6% for selective COX‐2 inhibitors with esomeprazole compared with selective COX‐2 inhibitors alone.^19^, ^20^ Our study also indicated a decreased risk of PUB for selective COX‐2 inhibitors with PPIs compared with selective COX‐2 inhibitors alone. However, the association was not significant. A possible explanation for this discrepancy is that our study included a relatively small number of patients exposed to selective COX‐2 inhibitors, leading to a limited statistical power.

Our study showed that age modified the risk of PUB for conventional NSAIDs with PPIs and selective COX‐2 inhibitors alone compared with conventional NSAIDs alone. Compared with younger adults, patients aged ≥75 years taking conventional NSAIDs with PPIs or apparently selective COX‐2 inhibitors with PPIs were associated with a lower risk of PUB, but those taking selective COX‐2 inhibitors alone had a higher risk with conventional NSAIDs alone as the comparator. These findings are consistent with several previous studies. A study conducted in France demonstrated that patients aged ≥60 years taking selective COX‐2 inhibitors alone had a higher rate of GI adverse events compared with younger patients by 0.54–0.96 and 0–0.23 per 1000 patients, respectively.^28^ Another study performed in Canada indicated that patients aged ≥75 years taking celecoxib with a PPI had a 42% lower risk of GI hospitalization compared with younger elderly. In contrast to our result for those aged ≥75 years taking conventional NSAIDs with PPIs, this Canadian study mentioned that this age group had a slightly higher risk of GI hospitalization by 4% compared with younger patients.^22^ This different risk might be due to differences in study design, sample size, and comparator used. It was a retrospective cohort study involving a large number of patients taking a combination of conventional NSAIDs and a PPI by almost 20 000 patients. They restricted the comparator to celecoxib, while our study took into account all selective COX‐2 inhibitors.

Finally, our study found that sex did not modify relative risks of PUB for all comparisons. Even though a meta‐analysis mentioned the risk of serious GI complications was higher in men than women exposed to conventional NSAIDs and/or selective COX‐2 inhibitors,^23^ a previous Dutch cohort study conducted in a similar setting showed that men and women taking these medications shared a similar risk of GI hospitalization.^29^

### Sensitivity analysis

In contrast to our main analysis, the sensitivity analysis surprisingly showed conventional NSAIDs with PPIs significantly increased the relative risk of PUB by 25% compared with conventional NSAIDs alone. This finding can be explained by channeling. Patients taking conventional NSAIDs alone are likely to discontinue or switch therapy because of GI adverse events.^30^ Subsequently, a PPI is more likely to be added or selective COX‐2 inhibitors are more likely to substitute conventional NSAIDs. It indicates that patients who discontinued conventional NSAIDs with PPIs and then switched to a more stomach protective strategy had a high risk of PUB.

### Strengths and limitations

The strength of this study is it was population‐based and used a large study population of about 80 000 conventional NSAIDs and/or selective COX‐2 inhibitors users for whom high‐quality data on hospitalizations and drugs dispensing information were extracted over a 15‐year period. The completeness and the accuracy of dispensing data in the Dutch PHARMO RLS database are high.^31^ By comparing the different strategies to lower risk of PUB when in need of a NSAID in one observational study, the relative effect estimates of these strategies are a better comparison than when these contrasts were evaluated separately.

As in all case–control studies using databases, we also considered several potential biases, namely, selection bias, information bias, and confounding. Selection bias is unlikely to happen because we limited our cases to first hospitalized patients for PUB. Hence, we specified our attention to a certain spectrum of disease, that is, severe cases.

Information bias includes misclassification of exposure, outcome, and confounding. We had no direct measure of patients' adherence to medications (including the exposures) because the Dutch PHARMO RLS is a database with a dispensing record of drugs. This database neither has records on OTC drug use. The use of OTC NSAIDs might lead to misclassification (underestimation) of the exposures. However, we expected its effect on the relative risk is minimal because in the Netherlands, OTC NSAIDs are commonly used for a short duration (1–7 days),^32^ while the risks of GI complication are significantly increased after 84 days of conventional NSAIDs exposure, except for indomethacin.^5^ However, indomethacin is not available as an OTC drug in the Netherlands.^27^ We could not either take into account OTC PPIs use, but our analysis showed that availability of PPIs as OTC drug had no significant impact on relative risk for users of conventional NSAIDs and a PPI. With regard to the outcome, the validity of diagnoses in this database is high as shown for pneumonia and cardiovascular (CV) diseases.^33^, ^34^

With regard to confounding, as we restricted our study into current users of conventional NSAIDs or selective COX‐2 inhibitors, we minimized confounding by indication. Although we adjusted for the most relevant potential confounders such as concomitant medications and history of drug use, we had no information on the history of GI ulcers, lifestyles (smoking status and alcohol consumption), *Helicobacter pylori* infection, and body mass index that are also prognostic factors of PUB. However, the proportions of these lifestyle factors and *H. pylori* infection were equally distributed among a Dutch population with or without GI symptoms using conventional NSAIDs and/or selective COX‐2 inhibitors as shown in earlier observational studies.^35^, ^36^, ^37^ We also tried to minimize confounding by the history of GI ulcers by considering past use of acid‐lowering drugs as a proxy. In addition, in our case–control study, we were not able to estimate the absolute risks that might be estimated in a cohort study.

### Clinical implications

Even though several guidelines have been established in order to prevent GI toxicity for patients with an increased risk of GI problems during NSAID exposure, >58% of those did not receive a gastroprotective strategy.^24^ Our findings may help to reassure physicians in their therapeutic decision to decrease the potential GI risk. We found that the risk differences between the three strategies to lower the risk of PUB were not statistically significant, but there are some indications that the gastroprotective strategy can be based on the degree of GI risk. When the risk increases, the order to implement a preventive strategy might be a conventional NSAID plus a PPI, a selective COX‐2 inhibitor alone, and a selective COX‐2 inhibitor plus a PPI. Obviously, the choice does not depend only on GI risk but also on potential CV problems. For the selective COX‐2 inhibitors, the increased risk of CV events has been clearly shown in clinical trials. Meanwhile, this is less clear for conventional NSAIDs, although several observational studies have shown that conventional NSAIDs probably also increase the risk of CV disease.^38^, ^39^

## Conclusions

Our study demonstrated that conventional NSAIDs combined with PPIs, selective COX‐2 inhibitors alone, or combined with a PPI were associated with a significantly decreased risk of PUB compared with conventional NSAIDs alone. Although in the same order the gastroprotective effect appeared to increase, the differences were not statistically significant. Compared with conventional NSAIDs alone, the risk for patients aged ≥75 years taking conventional NSAIDs with PPIs was lower, whereas for those taking selective COX‐2 inhibitors alone the risk was higher than younger patients. Both sex and availability of PPIs as OTC drug did not modify the risk of PUB.

## Conflict of Interest

The authors declare no conflict of interest.

**Key Points**

- In clinical practice, there is a substantial underuse of gastroprotective strategies in patients in need of a nonsteroidal anti‐inflammatory drug (NSAID) with an increased risk of gastrointestinal problems.
- No studies have been found directly comparing a selective cyclooxygenase‐2 (COX‐2) inhibitor and a conventional NSAID alone or combined with a proton pump inhibitor (PPI) for the risk of perforation, ulcers, or bleeding.
- In clinical practice, compared with conventional NSAIDs alone, there appears to be a trend that from conventional NSAID with a PPI, a selective COX‐2 inhibitor alone to a selective COX‐2 inhibitor with a PPI, there is an increasing gastroprotective effect.
- In patients aged above 75 years, the gastrointestinal protective effect of conventional NSAIDs plus PPIs and selective COX‐2 inhibitors appear to be higher than in younger patients, while for selective COX‐2 inhibitors, this is the other way around.

## Supporting information


**Table A1.** Sensitivity results for odds ratios for perforation, ulcers or bleeding (PUB) events among current users of conventional NSAIDs or selective COX‐2 inhibitors alone or combined with PPIs.
**Table A2.** Effect modification of availability of PPIs as OTC drug toward the association between conventional NSAIDs or selective COX‐2 inhibitors alone or combined with PPIs and the risk of perforation, ulcers, or bleeding (PUB).
