# Supplementary material for: Gastrointestinal toxicity among patients taking selective COX‐2 inhibitors or conventional NSAIDs, alone or combined with proton pump inhibitors: a case–control study
Source: Pharmacoepidemiol Drug Saf. 2017 Mar 31;26(10):1141–8. doi: 10.1002/pds.4183 (PMC5655916; doi:10.1002/pds.4183)
Supplement: Supplementary file 1 — Table A1. Sensitivity results for odds ratios for perforation, ulcers or bleeding (PUB) events among current users of conventional NSAIDs or selective COX‐2 inhibitors alone or combined with PPIs. Table A2. Effect modification of availability of PPIs as OTC drug toward the association between conventional NSAIDs or selective COX‐2 inhibitors alone or combined with PPIs and the risk of perforation, ulcers, or bleeding (PUB). [file PDS-26-1141-s001.docx]

Annex.

Table 1. Sensitivity results for odds ratios for perforation, ulcers or bleeding (PUB) events among current users of conventional NSAIDs or selective COX-2 inhibitors alone or combined with PPIs

| Exposure | Cases  (n = 4,823) | Controls  (n = 12,888) | Crude OR  (95% CI) | Adjusted OR^Ŧ^  (95% CI) |
| --- | --- | --- | --- | --- |
| Current use^(1)^, n (%) |  |  |  |  |
| - Conventional NSAIDs - PPIs | 3,181 (66.0) | 9,462 (73.4) | 1 | 1 |
| - Conventional NSAIDs + PPIs | 1,260 (26.1) | 2,243 (17.4) | 1.67 (1.54-1.81)* | 1.25 (1.13-1.38)* |
| - Selective COX-2 inhibitors - PPIs | 274 (5.7) | 902 (7.0) | 0.90 (0.79-1.04) | 0.85 (0.67-1.06) |
| - Selective COX-2 inhibitors + PPIs | 108 (2.2) | 281 (2.2) | 1.14 (0.91-1.43) | 0.84 (0.62-1.13) |

Abbreviations:

NSAIDs = non-steroidal anti-inflammatory drugs; COX-2 = cyclooxygenase-2; PPIs = proton-pump inhibitors; OR = odd ratio

^Ŧ^ Adjusted for age, sex, concomitant drugs (acid lowering drugs, vitamin K antagonists, platelet aggregation inhibitors, glucocorticoids and serotonin selective re-uptake inhibitors), and a history of drug use (conventional NSAIDs, selective COX-2 inhibitors and acid lowering drugs)

*statistically significant (p<0.05)

^(1)^ Patients who discontinued conventional NSAIDs or selective COX-2 inhibitors within 90 days prior to the index date or current use of these medications at the index date

Table 2 Effect modification of availability of PPIs as OTC drug toward the association between conventional NSAIDs or selective COX-2 inhibitors alone or combined with PPIs and the risk of perforation, ulcers, or bleeding (PUB)

|  | Cases | Controls | Crude OR  (95% CI) | Adjusted OR ^Ŧ^  (95% CI) | Crude SI  (95% CI) | Adjusted SI^Ŧ^  (95% CI) |
| --- | --- | --- | --- | --- | --- | --- |
| Not available , n (%) |  |  |  |  | 0.87 (0.56-1.36) | 0.88 (0.56-1.39) |
| - Conventional NSAIDs - PPIs | 222 (84.7) | 417 (87.2) | 1 | 1 |  |  |
| - Conventional NSAIDs + PPIs | 40 (15.3) | 61 (12.8) | 1.23 (0.80-1.90) | 0.90 (0.57-1.42) |  |  |
| Available, n (%) |  |  |  |  |  |  |
| - Conventional NSAIDs - PPIs | 1,377 (65.2) | 2,596 (66.7) | 1 | 1 |  |  |
| - Conventional NSAIDs + PPIs | 735 (34.8) | 1,295 (33.3) | 1.07 (0.45-2.58) | 0.79 (0.32-1.97) |  |  |

Abbreviations:

NSAIDs = non-steroidal anti-inflammatory drugs; COX-2 = cyclooxygenase-2; PPIs = proton-pump inhibitors; OR = odd ratio; SI = synergy index

**^Ŧ^** Adjusted for age, sex, concomitant drugs (acid lowering drugs, vitamin K antagonists, platelet aggregation inhibitors, glucocorticoids and selective serotonin receptor inhibitors), and a history of drugs use (conventional NSAIDs, selective COX-2 inhibitors and acid lowering drugs)

*statistically significant (p<0.05)
